# Vancomycin population pharmacokinetics and dosing proposal for the initial treatment in obese adult patients

**DOI:** 10.3389/fphar.2024.1364681

**Published:** 2024-06-04

**Authors:** Lucie Polášková, Irena Murínová, Jana Gregorová, Ondřej Slanař, Martin Šíma

**Affiliations:** ^1^ Department of Clinical Pharmacy, Military University Hospital Prague, Prague, Czechia; ^2^ Department of Applied Pharmacy, Faculty of Pharmacy, Masaryk University, Brno, Czechia; ^3^ Department of Clinical Pharmacy, Bulovka University Hospital, Prague, Czechia; ^4^ Department of Pharmacology, First Faculty of Medicine, Charles University and General University Hospital in Prague, Prague, Czechia

**Keywords:** clinical pharmacy, glomerular filtration rate, lean body mass, Monte Carlo simulation, non-linear mixed-effects modeling, obesity, therapeutic drug monitoring

## Abstract

**Aim:**

The aim of this study was to develop a vancomycin population pharmacokinetic model in adult obese patients and propose covariate-based dosing individualization in order to maximize the achievement of the newly recommended PK/PD target, according to a revised consensus guideline from 2020.

**Methods:**

Therapeutic drug monitoring data from initial vancomycin therapy (first 3 days of treatment) in adult obese (BMI ≥ 30 kg/m^2^) patients from 2013 to 2022 were analyzed using a non-linear mixed-effects modeling method, and Monte Carlo simulations were then used to find the optimal dosage maximizing the PK/PD target attainment.

**Results:**

A total of 147 vancomycin serum levels obtained from 138 patients were included in the analysis. Based on the covariate model diagnosis among all tested variables, no reliable predictor of vancomycin volume of distribution (Vd) was identified, while clearance (CL) was positively correlated with eGFR and lean body mass. Creatinine-based eGFR predicted vancomycin CL better than cystatin C-based eGFR. The median (interquartile range) value from conditional modes of individual estimates of Vd, CL, and elimination half-life in our population was 74.0 (70.5–75.4) L, 6.65 (4.95–8.42) L/h, and 7.7 (6.0–10.0) h, respectively.

**Conclusion:**

We proposed dosing individualization based on the covariate found in order to maximize the achievement of the newly recommended PK/PD target of the AUC/MIC ratio of 400–600. Clinical pharmacy/pharmacology interventions may lead to an improvement in vancomycin dosing with a reflection in PK/PD target attainment.

## 1 Introduction

Vancomycin is a glycopeptide antibiotic indicated for the treatment of patients with suspected or proven invasive Gram-positive infections, including methicillin-resistant *Staphylococcus aureus* (MRSA). Because it is a large compound with a molecular weight of approximately 1,450 Da, it is not absorbed efficiently after oral administration and must be administered intravenously to treat systemic infections (Rybak et al., 2009). After administration, vancomycin quickly penetrates various body fluids and tissues, with a volume of distribution (Vd) ranging from 0.4 to 1 L/kg (Rybak, 2006). The majority of vancomycin doses are excreted unchanged via glomerular filtration, with an elimination half-life (t_1/2_) of 6–12 h, which can be significantly prolonged in patients with renal insufficiency (Rybak, 2006). Therefore, vancomycin dosing must be adjusted according to renal functional status (Sima et al., 2018b). The ratio of the 24-h area under the concentration-time profile (AUC_24_) to the minimal inhibitory concentration (MIC) of ≥400 was associated with a successful outcome in patients with MRSA pneumonia (Moise-Broder et al., 2004), and therefore, this value was generally considered the most appropriate pharmacokinetic/pharmacodynamics (PK/PD) parameter for vancomycin treatment. To achieve this PK/PD target and prevent vancomycin toxicity, therapeutic drug monitoring (TDM) was traditionally used in widespread clinical practice when promoting trough vancomycin levels in the range of 15–20 mg/L as a surrogate marker for optimal vancomycin exposure (Rybak et al., 2009). However, in light of new studies showing, for example, that many patients with vancomycin trough levels in the range of 15–20 mg/L were overexposed, a revised consensus guideline by the American Society of Health-System Pharmacists, the Infectious Diseases Society of America, the Pediatric Infectious Diseases Society, and the Society of Infectious Diseases Pharmacists was published in 2020 (Rybak et al., 2020). This guideline strongly recommends using the direct PK/PD target of an AUC_24_ of 400–600 mg h/L (assuming a MIC of 1 mg/L) instead of trough levels of 15–20 mg/L.

Appropriate vancomycin exposure should be achieved early during the course of therapy, preferably within the first 24–48 h. In clinical routines, it is common practice to measure vancomycin concentrations after reaching the expected steady state, i.e., usually prior to the fourth or fifth dose. Therefore, most studies addressing the PK and PK-based dosing of vancomycin in clinical practice focus on maintenance dosing in steady-state, while data describing the initial phase of pharmacotherapy are lacking.

Obesity, defined as a body mass index (BMI) ≥ 30 kg/m^2^, is often attributed to weight gain caused by increased deposition of adipose tissue, but obesity also involves a number of physiological changes, including increases in muscle mass and connective tissue (Craig, 1998). The hydrophilicity of vancomycin, together with the increase in adipose tissue and muscle mass in obesity, is likely to lead to greater variability in Vd compared with non-obese patients (Grace, 2012). In addition, increased blood flow caused by the increased cardiac output and blood volume in obese patients may also contribute to this phenomenon (Smit et al., 2018). Overweight and obese patients also often exhibit augmented glomerular filtration rate and renal plasma flow, which is attributed to compensatory renal vasodilation overcoming increased tubular sodium reabsorption (Chagnac et al., 2000).

These three points—i.e., PK/PD target correction, lack of data for the initial phase of therapy, and possible changes in PK in obesity—justify the need to address the initial dosing of vancomycin in obese patients. Therefore, the aim of this study was to develop a vancomycin population PK model based on TDM data during initial treatment in adult obese patients and propose covariate-based dosing individualization, maximizing the achievement of the newly recommended vancomycin PK/PD target by a revised consensus guideline from 2020.

## 2 Methods

### 2.1 Study design

An open-label retrospective observational study was performed on obese adult patients (age ≥18 years; body mass index, BMI ≥30 kg/m^2^) treated with vancomycin intravenous infusion admitted to mixed wards of the Military University Hospital in Prague from January 2013 to December 2022. Patients with at least one measured vancomycin serum concentration during initial therapy (the first 3 days of treatment) were included. The exclusion criteria were extracorporeal life support and renal replacement therapy. The study was approved by the local Ethics Committee of the Military University Hospital in Prague under registration number 108/17-42/2022 and followed the principles laid down in the Declaration of Helsinki. Since the retrospective nature of this study involved only the analysis of routine clinical data, study-specific informed consent was waived. The collection of anonymized data and its processing are in the public interest.

### 2.2 Data retrieval

The clinical records of all evaluated patients were reviewed to collect information concerning age, gender, body weight, height, and serum levels of creatinine and urea. If available, the serum cystatin C level was also collected. The body mass index (BMI) was calculated as body weight (kg) divided by the square of height (m). The body surface area (BSA) and lean body mass (LBM) were calculated using Du Bois and Boer formulas, respectively (Boer, 1984; Du Bois and Du Bois, 1989). Both creatinine- and cystatin C-based (if available) glomerular filtration rates (eGFR) were estimated according to the Chronic Kidney Disease Epidemiology Collaboration (CKD-EPI) formula for each patient (Inker et al., 2012). The vancomycin-dosing regimen, including administration times and infusion rates, was recorded. Vancomycin serum concentrations were determined as a routine part of the TDM monitoring procedure at the Department of Clinical Biochemistry of the Military University Hospital in Prague, and sampling times were also recorded. Vancomycin levels in the serum were determined using the immunoturbidimetric method based on the kinetic interaction of microparticles in solution (KIMS) on the Roche Cobas 8,000 Analyzer (Roche, Basel, Switzerland). The lower limits of detection and quantitation were 1.5 and 4.0 mg/L, respectively, and the measuring range was from 4.0 to 80.0 mg/L. Only levels measured during initial vancomycin therapy (first 3 days of treatment) were included in the PK analysis. If available, the MIC value of vancomycin for the isolated bacterial strain was also recorded. All microbiological samples were processed at the Department of Clinical Microbiology of the Military University Hospital in Prague. Antimicrobial susceptibility testing was performed using the disc diffusion method and broth microdilution method. Results were interpreted according to the European Committee on Antimicrobial Susceptibility Testing (European Committee on Antimicrobial Susceptibility Testing, 2022).

### 2.3 Population PK analysis

Serum concentration–time profiles of vancomycin were analyzed using the non-linear mixed-effects modeling method. The model parameters were assumed to be log-normally distributed and were estimated by maximum likelihood using the stochastic approximation expectation maximization (SAEM) algorithm within MonolixSuite software version 2021R2 (Lixoft SAS, Antony, France).

For the structural model, one- and two-compartment models with first-order and Michaelis–Menten elimination kinetics were tested. Log-normally distributed inter-individual variability terms with estimated variance were tested on each PK parameter. Constant, proportional, and combined error models were tested for the residual error model. The most appropriate model was selected based on the objective function value (OFV), Akaike information criterion (AIC), Bayesian information criterion (BIC) differences, adequacy of the goodness-of-fit (GOF) plots, and low relative standard errors (R.S.Es) of the estimated PK parameters.

Age, BW, height, LBM, BSA, BMI, serum creatinine, urea, and eGFR were tested as continuous covariates, while gender and diagnosis were tested as categorical covariates of PK parameters. A preliminary graphical assessment and univariate association using Pearson’s correlation test of the effects of covariates on estimated PK parameters were made. The covariates with *p* < 0.05 were considered for the covariate model. Afterward, a stepwise covariate modeling procedure was performed. For model selection, forward addition and backward elimination methods were used. In the forward addition, a decrease in the OFV of more than 3.84 points between nested models (*p* < 0.05) was considered statistically significant, assuming a χ^2^ distribution. In backward elimination, covariates were retained in the model if the difference in the OFV was greater than 6.64 points between nested models (*p* < 0.01). Additional criteria for model selection were reasonably low R.S.E. values of the estimates of model parameters, the physiological plausibility of the obtained parameter values and the covariates found, and the absence of bias in GOF plots.

Model adequacy was evaluated using GOF plots. For both the structural (non-covariate) model and final covariate model, observed concentrations were plotted against individual and population predictions; the population-weighted residuals (PWRES) were plotted against time and population predictions; and the normalized prediction distribution errors (NPDEs) were plotted against time and population predictions. To evaluate the stability of the model, a bootstrap analysis was performed. In this procedure, 250 replicates of the original data were generated, and the parameter estimates for each of the 250 samples were re-estimated using the R package Rsmlx for MonolixSuite (Lixoft SAS, Antony, France) in the final model. The median and 95% confidence intervals (CI) obtained for each parameter estimated for bootstrap samples were compared with the estimates in the final model.

### 2.4 Monte Carlo simulations

Monte Carlo simulations (250 replicates of the original dataset) based on the final population PK model were performed to generate the theoretical distribution of areas under the vancomycin concentration–time curves (AUC) during initial treatment (first 60 h) using SimulX version 2021 (Lixoft SAS, Antony, France). The whole study population was stratified according to the covariates found (eGFR of < 0.5, 0.5–1, 1–1.5, and 1.5–2.13 and > 2.13 mL/s/1.73 m^2^, and LBM of < 70 and > 70 kg), and the administration of various vancomycin dosing regimens was simulated in each subpopulation. Considering a usual MIC of 1 mg/L, vancomycin AUC over 24 h of 400–600 mg h/L was elected as an optimal PK/PD target (Rybak et al., 2020), and a vancomycin dosing regimen showing the highest probability of the target attainment was identified for each subpopulation.

### 2.5 Calculations and statistics

Vancomycin t_1/2_ was calculated from Vd and clearance (CL) values using the following formula: t_1/2_ = 0.693 × Vd/CL. Descriptive parameter medians and interquartile range (IQR) were calculated using MS Excel 2013 (Microsoft Corporation, Redmond, United States). The Mann–Whitney U-test was used to compare vancomycin loading doses (LDs)with and without the guidance of clinical pharmacists and compare trough levels reached after loading dose administration. The linear regression model was used to evaluate the relationships between vancomycin PK parameters (Vd and CL) and continuous variables (BW, LBM, BSA, BMI, creatinine, and cystatin C-based eGFR). GraphPad Prism software version 8.2.1 (GraphPad Inc., La Jolla, CA, USA) was used for all comparisons, and *p*-levels <0.05 were considered statistically significant.

## 3 Results

### 3.1 Study population

A total of 138 patients (82 males and 56 females) were enrolled in the study. Their demographic and laboratory characteristics are summarized in Table 1. There was a broad spectrum of infection severities and origins. Patients received vancomycin to treat infections of the central nervous system (*n* = 46; 33%), sepsis (*n* = 25; 18%), orthopedic (*n* = 21; 15%), ocular (*n* = 12; 9%), skin (*n* = 8; 6%), or other infections (*n* = 26; 19%), such as pneumonia, bacteriuria, bacteremia, endocarditis, or intra-abdominal infection. The most frequent infectious pathogens in this study were various strains of *Staphylococcus* spp. (*aureus*, *haemolyticus*, *hominis*, *capitis*, *epidermidis*, and *warneri*), *Enterococcus* spp. *(faecalis*, *faecium*, and *cloacae*), *Klebsiella pneumoniae*, and *Streptococcus agalactiae*. The median (min–max) and modus values of the MIC were 1 (≤0.5–2) and 1 mg/L, respectively.

**TABLE 1:** Demographic and laboratory data of the patients (*n* = 138).

Characteristic	Median	IQR	Range
Age (years)	65	54–72	26–86
Body weight (kg)	104	95–120	73–190
Height (m)	1.74	1.65–1.80	1.50–1.95
Lean body mass (kg)	68	55–76	41–104
Body surface area (m^2^)	2.17	2.02–2.35	1.68–2.77
Body mass index (kg/m^2^)	34.3	32.5–38.3	30.1–65.7
Serum creatinine (μmol/L)	72.3	55.8–98.1	23.2–374.1
Serum urea (mmol/L)	5.6	3.8–7.9	1.2–24.4
eGFR (mL/s/1.73 m^2^)	1.51	1.12–1.72	0.17–2.47

eGFR, glomerular filtration rate estimated according to CKD-EPI formula; IQR, interquartile range.

**FIGURE 1:**
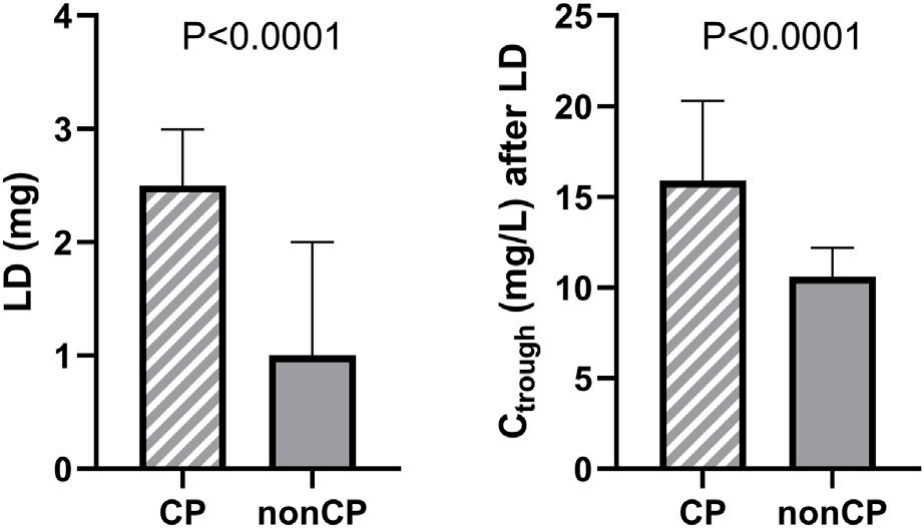
Comparison of vancomycin LDs administered with and without the guidance of clinical pharmacists (CP) and comparison of vancomycin trough levels (C_trough_) reached after these LD administrations.

A vancomycin LD of 1–4 g (median 2.5 g) via a 0.5–7-h (median 5-h) intravenous infusion was administered to 122 patients. A maintenance dose (MD) ranging from 0.5 to 1.5 g (median 1 g) was administered every 6, 8, 12, or 24 h via a 1–3-h (median 2-h) intravenous infusion. In nine cases, vancomycin MD was administered as a continuous infusion of 1–4 g (median 2 g) per day. LDs administered on the advice of clinical pharmacists were significantly higher than those administered without consulting this service, which was subsequently mirrored in the significant difference in trough levels achieved after these LDs (Figure 1). Median trough levels after LD with and without the guidance of clinical pharmacists were in and out of the recommended therapeutic range for vancomycin troughs (15–20 mg/L), respectively (Rybak et al., 2020).

A total of 147 vancomycin serum concentrations were included in the PK analysis (1–2 per patient), where 11 concentration points (7.5%) were taken as a peak (sample collection up to 2 h after infusion completion), 124 concentration points (84.4%) were taken as a trough level (0–1 h before the next dose is administered), and 12 samples (8.2%) were taken in the course of the dosing interval (with an exact record of the time of sampling).

### 3.2 Population pharmacokinetic analysis

A one-compartment model with linear elimination kinetics best-fitted vancomycin concentration–time data. A constant error model was the most accurate for the description of residual and interpatient variability. The PK model was parametrized in terms of Vd and CL.

**FIGURE 2:**
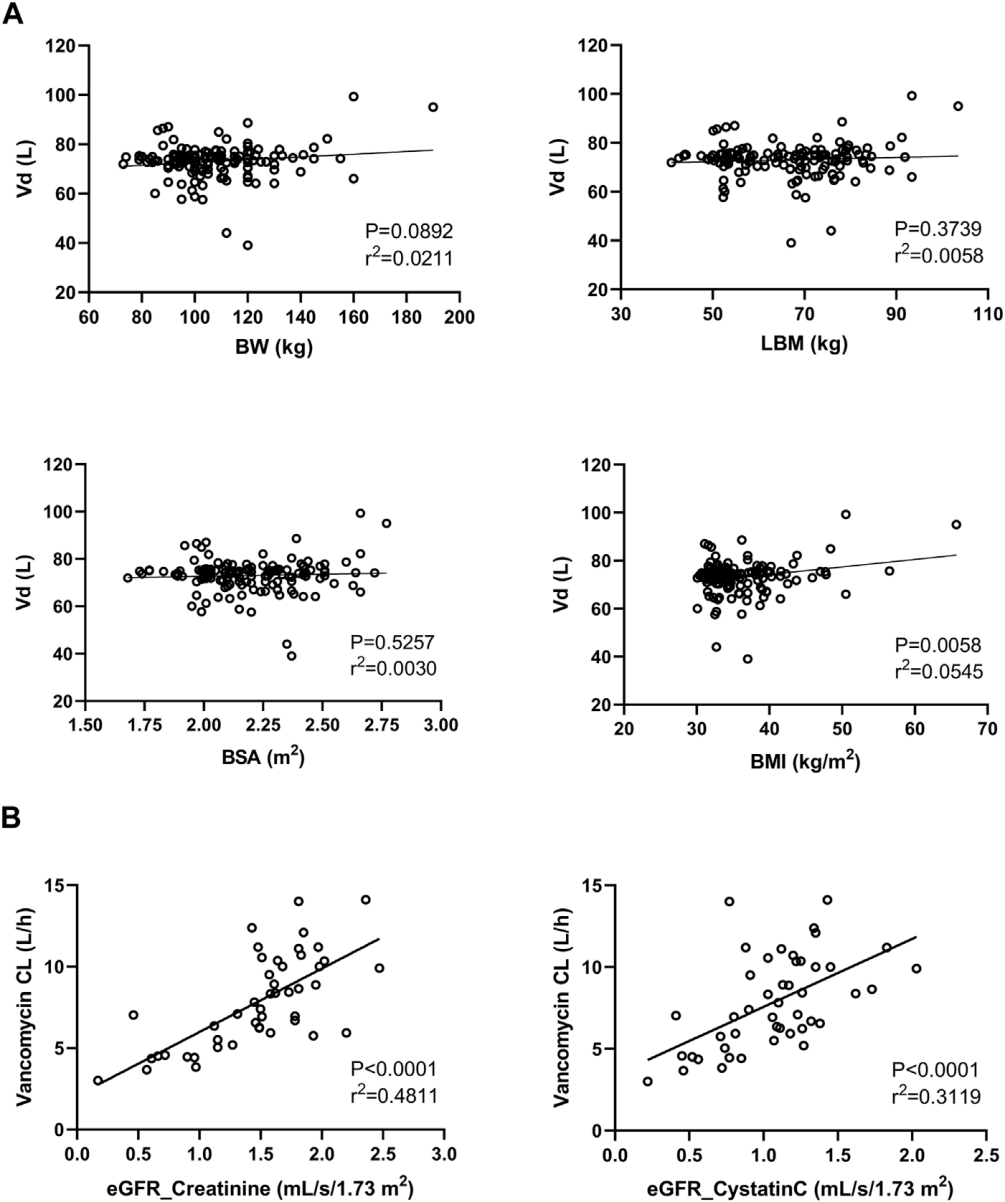
Relationship between the vancomycin volume of distribution (Vd) and body size descriptors (BW, body weight; LBM, lean body mass; BSA, body surface area; and BMI, body mass index) **(A)**. Relationship between vancomycin clearance (CL) and both creatinine- and cystatin C-based estimations of the glomerular filtration rate **(B)**.

The preliminary graphical assessment showed only a very weak relationship between BMI and vancomycin Vd, whereas the other body size descriptors (BW, LBM, and BSA) were found to be without statistical significance (Figure 2A), which is reflected in the final model, where covariate diagnostics found that none of the covariates tested reliably predicted vancomycin Vd. Based on a preliminary graphical assessment, vancomycin CL was positively related to eGFR, BW, height, LBM, and BSA and negatively related to serum creatinine and urea. Covariate model diagnostics showed that among all tested variables, vancomycin CL was best predicted using eGFR and LBM. In a subgroup of patients in whom cystatin C was also measured (*n* = 46), we compared the predictive performance of both creatinine- and cystatin C-based eGFR, with creatinine-based eGFR proved to be a better predictor of vancomycin CL in this case (Figure 2B).

The population PK estimates and bootstrap results in the final population model are summarized in Table 2.

**TABLE 2:** Estimates of the final vancomycin population pharmacokinetic model and bootstrap results based on 250 simulations.

Parameter	Final model		Bootstrap analysis
	Estimate	R.S.E. (%)	Median (95% CI)
*Fixed effects*			
Vd_pop (L)	75.0	8.66	79.3 (77.8–80.9)
CL_pop (L/h)	1.32	19.3	1.27 (1.24–1.31)
β_CL_LBM (kg)	0.011	21.6	0.011 (0.0108–0.0113)
β_CL_eGFR (mL/s/1.73 m^2^)	0.61	11.8	0.61 (0.60–0.63)
*Standard deviation of the random effects*			
Ω_Vd	0.31	16.6	0.38 (0.37–0.38)
Ω_CL	0.28	9.92	0.29 (0.28–0.29)
*Error model parameters*			
Constant	2.9	20.7	2.2 (2.1–2.3)

Vd, volume of distribution; CL, clearance; LBM, lean body mass; eGFR, estimated glomerular filtration rate; CI, confidence interval; R.S.E., relative standard error; pop, typical value of parameter; β, covariate effect on parameter; and Ω, standard deviation of the random effects.

The final equations describing the relationships between vancomycin PK parameters and their covariates are as follows:

Vd=Vd_pop,

$$CL=CL\_pop\times e^{\beta \_CL\_eGFR\times eGFR}\times e^{\beta \_CL\_LBM\times LBM},$$

where pop represents the typical value of the parameter, β represents the covariate effect on the parameter, LBM is the lean body mass according to the Boer formula, and eGFR is the estimated glomerular filtration rate according to the creatinine-based CKD-EPI formula.

In our study population, the median (interquartile range) values from individual estimates of Vd, CL, and t_1/2_ expressed as conditional modes were 74.0 (70.5–75.4) L, 6.65 (4.95–8.42) L/h, and 7.7 (6.0–10.0) h, respectively.

**FIGURE 3:**
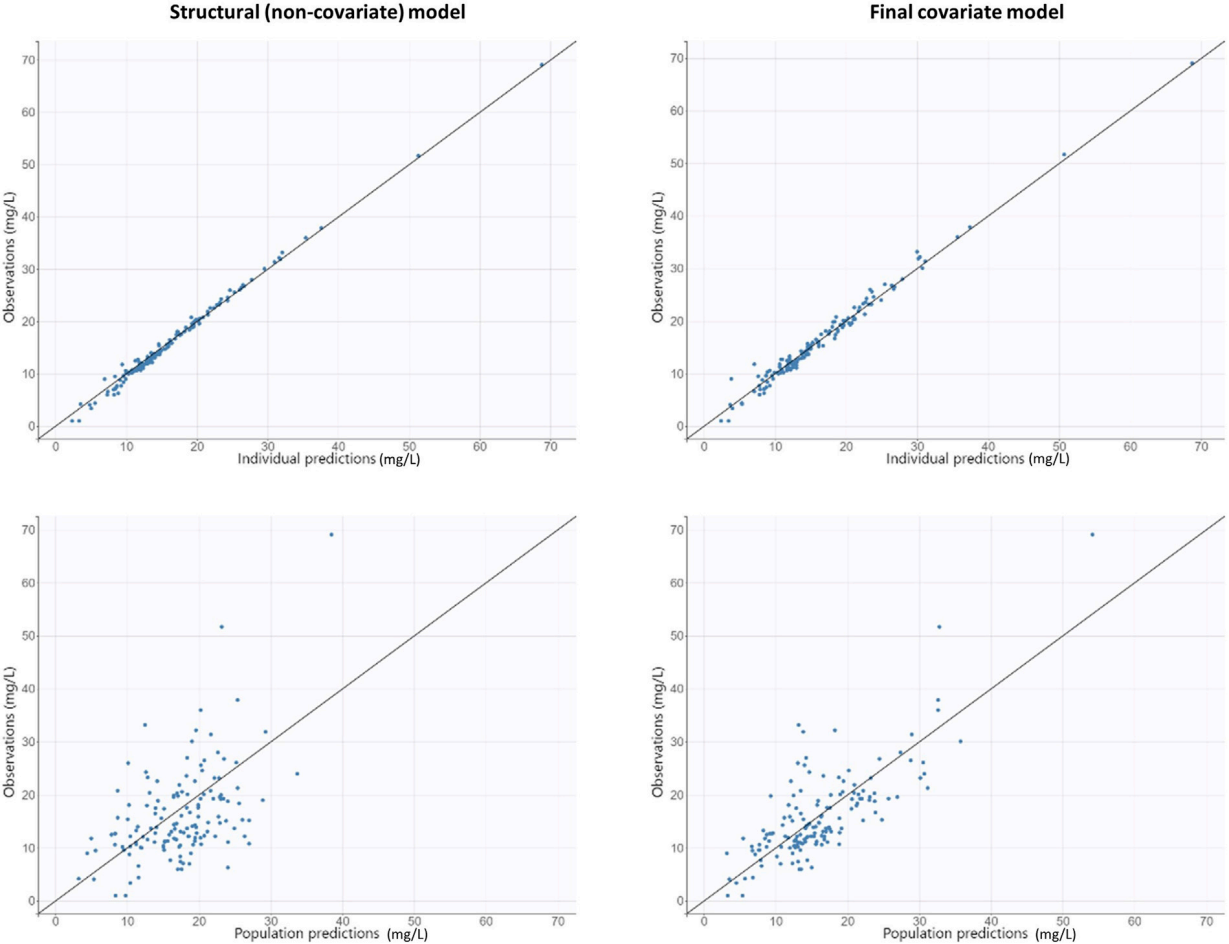
Goodness-of-fit plots obtained from both the structural (non-covariate) and final covariate models for vancomycin: population and individual predictions against observed concentrations.

**FIGURE 4:**
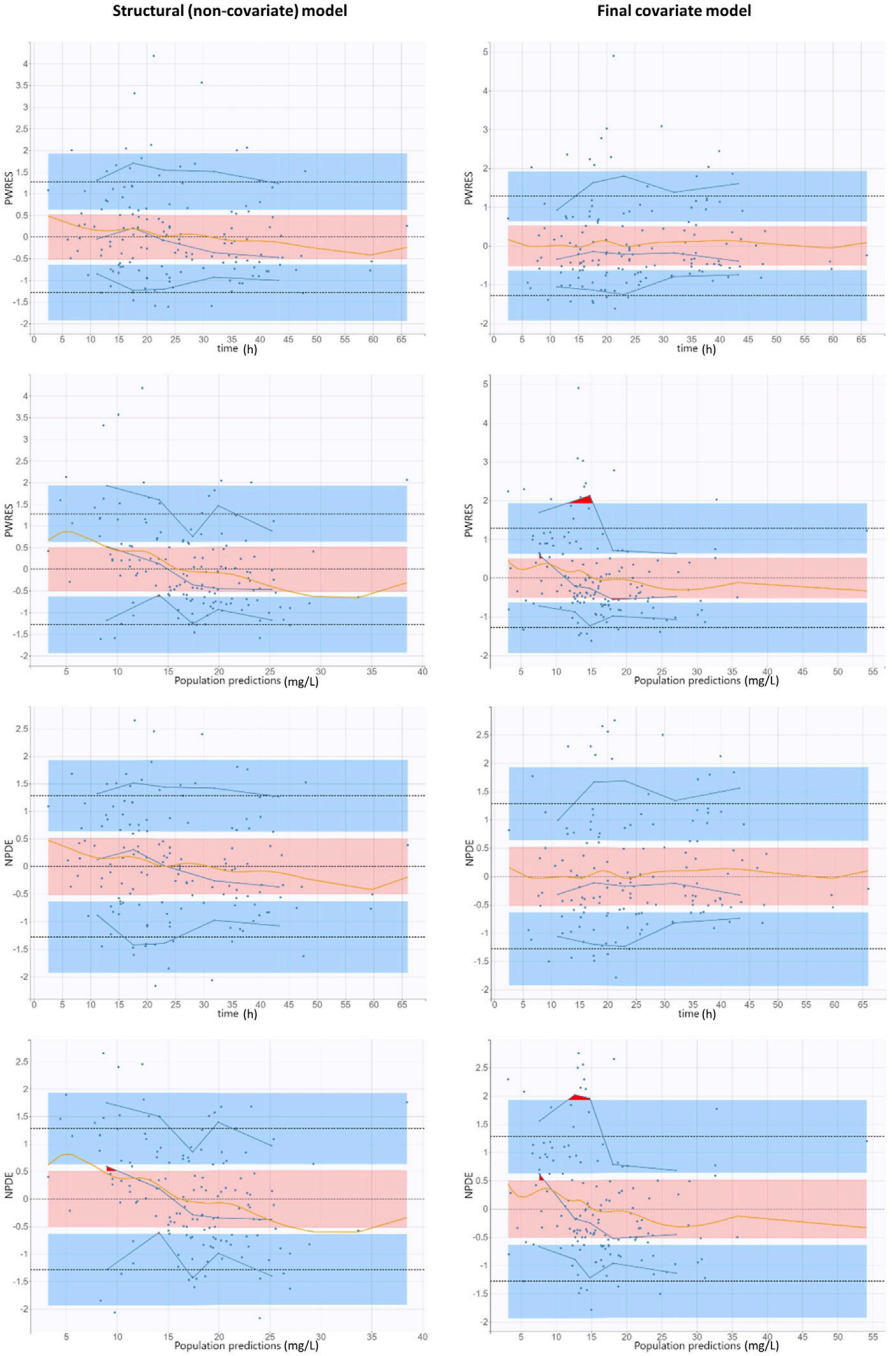
Goodness-of-fit plots obtained from both the structural (non-covariate) and final covariate models for vancomycin: population-weighted residuals and normalized prediction distribution errors versus time after vancomycin dose and versus population predictions. Solid blue lines represent the 10th, 50th, and 90th percentiles of the observed data. Shaded regions represent 95% confidence interval around the 10th (below blue region), 50th (pink region), and 90th (above blue region) percentiles of the simulated data. The observations are represented by blue dots. Orange curves represent smoothing splines. Outliers (empirical percentiles outside the prediction intervals) are marked with red areas.

The diagnostic GOF plots for the final covariate model did not indicate major deviations (Figures 3, 4). As shown in Table 2, the R.S.E. (maximum 21.6%) revealed that all PK parameters in the model were precisely estimated. All median parameter values in the bootstrap procedure were consistent with the values obtained in the final model fit, indicating the reliability of the parameter and the random-effect estimates.

### 3.3 Monte Carlo simulations

Table 3 summarizes the covariate-based dosing individualization of vancomycin in obese patients proposed based on Monte Carlo simulations (250 replicates of the original dataset, i.e., 34,500 simulated PK profiles) in order to maximize the probability of target attainment when AUC over 24 h of 400–600 mg h/L was considered a PK/PD target. Using the proposed posology, the overall probability of the target attainment was 57.7% in the whole population. For comparison, when we simulated the administration of vancomycin LD at 2,000 mg followed by the MD at 1,000 mg every 8 h (the most frequent initial dosage used in our real population), the probability of the target attainment was only 44.5%.

**TABLE 3:** Proposed individualization of vancomycin dosing in obese patients in order to maximize the probability of target attainment (AUC_24_ of 400–600 mg h/L).

eGFR (mL/s/1.73 m^2^)	LBM (kg)	Dosing (h)	PTA (%)
< 0.5	< 70	LD 2000 mg + MD 750 mg every 12	67.5
	> 70	LD 2000 mg + MD 750 mg every 8	55.0
0.5–1	< 70	LD 2000 mg + MD 750 mg every 8	60.9
	> 70	LD 2000 mg + MD 750 mg every 8	62.2
1–1.5	< 70	LD 2500 mg + MD 1000 mg every 8	53.6
	> 70	LD 2500 mg + MD 1000 mg every 8	57.5
1.5–2.13	< 70	LD 2500 mg + MD 1250 mg every 8	55.9
	> 70	LD 2500 mg + MD 1500 mg every 8	58.3
> 2.13	< 70	LD 3000 mg + MD 1250 mg every 6	59.5
	> 70	LD 3000 mg + MD 1500 mg every 6	57.0

eGFR, estimated glomerular filtration rate; LBM, lean body mass; PTA, probability of target attainment; LD, loading dose; and MD, maintenance dose.

## 4 Discussion

The World Health Organization reported the current obesity prevalence of more than 20% in adult inhabitants of the American and European regions and warned of an ever-increasing trend toward obesity (Boutari and Mantzoros, 2022). The need for appropriate dosing for obese patients will therefore inevitably be encountered with increasing frequency. Body weight gain in obese patients, accompanied by physiological changes, leads to alterations in drug pharmacokinetics, and thus, dosage adjustments in a weight-proportional manner may not be accurate. This is evidenced by studies in which higher trough concentrations of vancomycin were observed in overweight and obese patients in comparison with non-obese patients when dosing was based on total body weight (Miller et al., 2011; Heble et al., 2013). On the other hand, the administration of LDs is often omitted during vancomycin therapy in routine clinical practice, which can lead to significant drug underexposure at the beginning of the treatment. The situation is further complicated by the change in vancomycin TDM recommendations from trough-based to AUC_24_-based targeting of therapy, which requires either sampling multiple levels or evaluating AUC_24_ using software with Bayesian estimations. As shown in Figure 1, this issue can significantly improve the availability of clinical pharmacy/pharmacology services. It is important to note, however, that in order to set up vancomycin therapy correctly from the beginning, this consultation service must be requested before starting the treatment and not just for the interpretation of vancomycin levels after its measurement, as is usually the practice. Still, there is a need for an appropriate tool to optimize initial vancomycin therapy in the obese. Therefore, we decided to develop a population PK model focusing on the initial phase of therapy in this specific cohort.

Our PK model best fits concentration–time data using a one-compartment structure. Although this is consistent with findings in some other studies (Adane et al., 2015; Hong et al., 2015), a two-compartment arrangement is generally assumed in vancomycin (Sima et al., 2019). Vancomycin distribution half-life is approximately 7 min, and therefore, the distribution phase duration is approximately 0.5 h (Matzke et al., 1986). Since peak levels were drawn just after the completion of the vancomycin infusion (minimally 1 h), this sampling strategy could not capture the distribution phase. However, since it was described that the distribution phase contributes only negligibly to total vancomycin exposure (<10%) (Shingde et al., 2019), the use of the one-compartment model should not have a clinically relevant impact on the estimation of the PK/PD target attainment.

A typical value of vancomycin population Vd was 75 L in our study, and none of the tested variables was found to be its covariate (Figure 2A). This may seem to be a surprising finding, as it is generally accepted that vancomycin Vd is proportional to body weight (Rybak et al., 2020). However, it is physiologically plausible that the Vd of hydrophilic compounds does not increase proportionally with body weight in obese patients, in whom weight gain is mainly due to the deposition of adipose tissue. This phenomenon leads to high variability, which can overlay the relationship between Vd and body weight. Moreover, some other studies in obese patients corroborate our finding with weight-independent estimates of vancomycin Vd (Hong et al., 2015; Zhang et al., 2023).

In our population model, eGFR and LBM were identified as the most appropriate covariates of vancomycin CL based on the maximum reduction in OFV, RSE, and unexplained variability of this parameter. Exactly, vancomycin CL was calculated as follows: $\text{CL}=1.32\times e^{0.61\times \text{eGFR}}\times e^{0.011\times \text{LBM}}$ . This means that, for example, in a patient with an LBM of 68 kg and an eGFR of 1.51 mL/s/1.73 m^2^ (median values in our study population), the CL of vancomycin would be estimated to be 7 L/h, which with a Vd of 75 L corresponds to a t_1/2_ of 7.4 h that is fully consistent with the evidence in the literature (Rybak, 2006). Generally, eGFR and body weight are the most commonly referred covariates of vancomycin CL (Ducharme et al., 1994). It is worth mentioning that the Cockroft–Gault equation, a frequently used equation for calculating eGFR, is not suitable for obese patients, as it includes body weight in the calculation and thus could often lead to a false overestimation of eGFR. For this reason, we used the CKD-EPI equation, whose superiority in the estimation of vancomycin CL has been demonstrated (Sima et al., 2018b). Similarly, alternative weight descriptors such as ideal body weight, adjusted body weight, fat-free weight, and LBW are often used for the estimation of PK parameters in hydrophilic drugs in order to prevent overexposure in obese patients (Pai, 2012; Sima et al., 2018a). Another perhaps somewhat surprising finding was that vancomycin CL was better predicted using creatinine than cystatin C (Figure 2B).

Although cystatin C is recommended for measuring eGFR when creatinine-based estimates are not considered sufficiently accurate (Carrero et al., 2023) and is often described as superior for estimating the CL of drugs excreted via the kidney (Sima et al., 2017), it can be falsely elevated in obese patients (Carrero et al., 2023). On the other hand, low creatinine production in cachectic patients, which falsely overestimates creatinine-based eGFR, is not very relevant for obese patients.

Based on the final covariate PK model, we proposed the individualization of vancomycin dosing based on eGFR and LBM in order to improve the proportion of patients achieving the newly recommended PK/PD target of AUC_24_ of 400–600 mg h/L. Vancomycin posology at the beginning of the treatment should consist of both LD and MD administration. Traditionally, MD is derived from the CL of the drug, while the LD is calculated from its Vd. However, it is important to note that Vd only determines the peak level at the end of the distribution phase after drug administration, while for the AUC-targeted dosing, CL is the primary parameter determining the achievement of the PK/PD target. Therefore, although we did not observe any covariates of Vd in our study, our dosing proposal uses eGFR and LBM as covariates of CL to individualize both MD and LD (Table 3). Total daily doses in each eGFR category may appear to be higher than generally reported for normal-weight patients. However, this is consistent with the increase in total drug CL that is often described in obese patients (while a similar body weight-normalized CL is usually described) (Gouju and Legeay, 2023). The higher drug CL in obese patients can also be explained by some physiological changes during obesity, such as increased blood flow, activation of the renin–angiotensin system, and glomerular hyperfiltration (Gouju and Legeay, 2023). Dosing recommendations for vancomycin usually end with an eGFR category of ≥1.5 mL/s/1.73 m^2^. Since 52% of the patients in our study had an eGFR ≥1.5 mL/s/1.73 m^2^ and 5% of these even had ≥2.13 mL/s/1.73 m^2^, we were able to suggest individualization of vancomycin dosing for patients with increased and augmented renal CL, in whom very high daily doses are needed to achieve the PK/PD target. Using the proposed posology, the overall probability of target attainment was 57.7%, which may not seem enough. Nevertheless, for example, at the most commonly used dosage of LD 2000 mg + MD 1000 mg every 8 h, the probability of target attainment would be only 44.5%. It is important to recognize that vancomycin is a drug with highly variable pharmacokinetics and that the suggested dosing is only for initial treatment prior to the measurement of vancomycin levels (no later than the third day of therapy). Thereafter, dosing must, of course, be adjusted and guided using TDM.

We acknowledge several slight limitations arising from the retrospective nature of the study and the fact that we only assessed PK/PD target achievement and not clinical outcomes. Furthermore, the dosage is valid only under the assumption that the MIC of 1 mg/L is the most common value for staphylococcal infections. In the case of targeting different MIC values, doses need to be adjusted. On the other hand, MIC values are typically not available within the first 3 days of therapy in clinical routines, yet current evidence indicates that the vancomycin PK/PD target needs to be optimized early in the course of infection (Rybak et al., 2020). Therefore, targeting and maintaining the AUC_24_ values between 400 and 600 mg h/L is a generally accepted and widely used practice for the adjustment of vancomycin initial treatment.

## 5 Conclusion

We developed a vancomycin population PK model in adult obese patients, where eGFR and LBM were found to be the most predictive covariates of vancomycin CL. This covariate-based dosing individualization was proposed in order to maximize the achievement of the newly recommended PK/PD target according to a revised consensus guideline from 2020. Clinical pharmacy/pharmacology interventions may lead to an improvement in vancomycin dosing with reflection in PK/PD target attainment.

## Data Availability

The raw data supporting the conclusion of this article will be made available by the authors, without undue reservation.
